# Supplementary material for: Spatially heterogeneous acetylcholine dynamics in the striatum promote behavioral flexibility
Source: Nat Commun. 2025 Dec 17;16:10877. doi: 10.1038/s41467-025-66826-1 (PMC12711930; doi:10.1038/s41467-025-66826-1)
Supplement: Supplementary file 1 — Supplementary Information [file 41467_2025_66826_MOESM1_ESM.pdf]

Spatially heterogeneous acetylcholine dynamics in the striatum promote behavioral flexibility

Sarpong *et al* – Supplementary Information

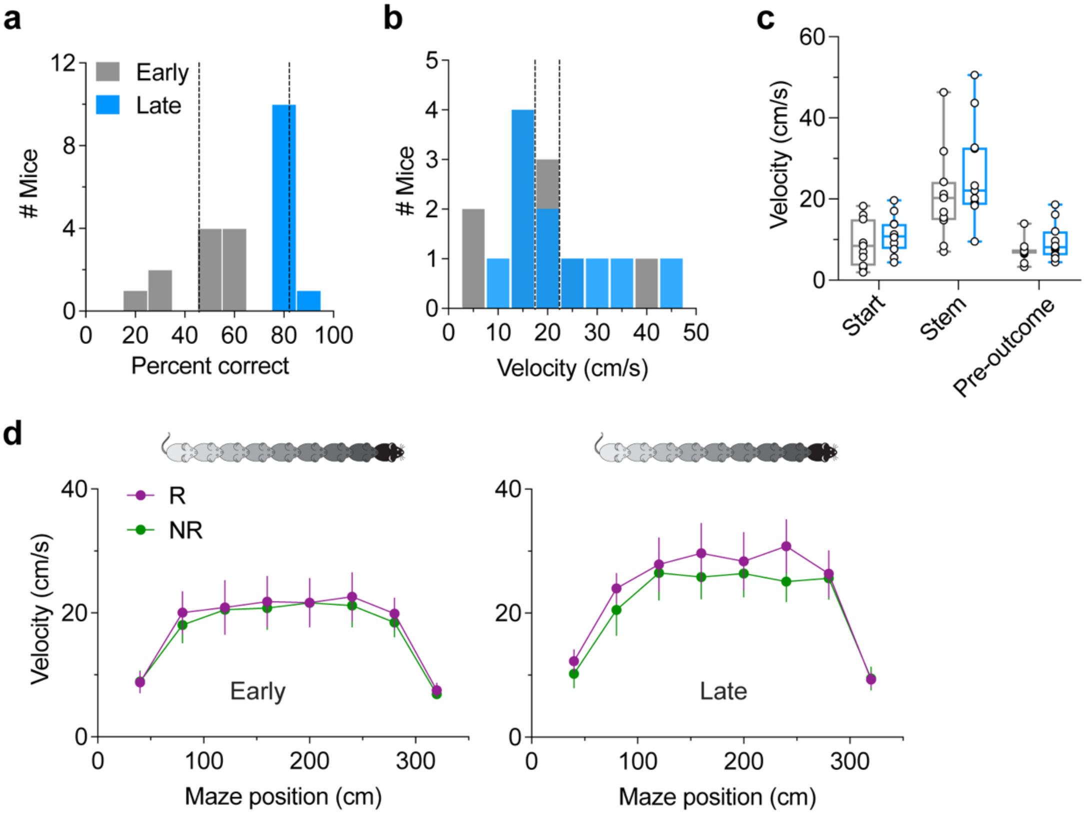

Supplementary Fig. 1: Adaptive changes in choice performance and running velocity across learning. a,**b**, Distribution of percentage correct choices (**a**) and velocity (**b**) of all recorded mice in the early (gray) and late (blue) phases of task acquisition (n = 11 mice). Mean performance was slightly below chance levels in the early sessions and improved to ≥80% correct choices by the last session. Dashed black line, median. **c**, Box plot showing the velocity (cm/s) of all mice in the early and late phases of task acquisition. Note the increases in velocity in the different zones of the virtual Y-maze (start, stem and pre-outcome). **d**, Velocity (mean ± S.E.M) as a function of mouse position (0-320 cm, 40-cm bins) in the maze during the early (left) and late phases (right) of task acquisition. In late sessions, mice tended to run faster to the rewarded arm relative to the no-reward arm. In box plots, center lines depict the median, box limits represent the 25th and 75th percentiles, and whiskers, data range. Source data are provided as a Source Data file.

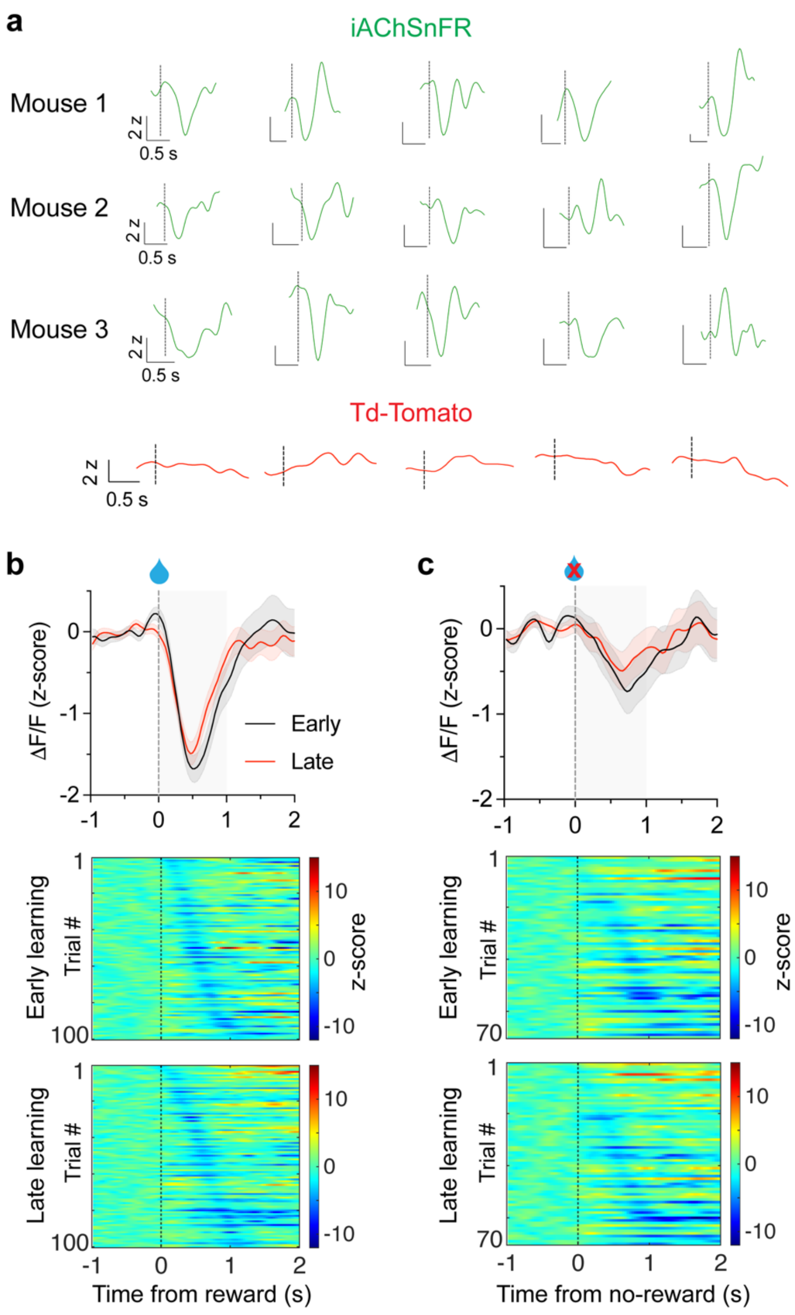

Supplementary Fig. 2: Dynamic changes in ACh responses during task acquisition. a, Example ACh responses in 3 representative mice showing trial-by-trial variability in reward responses. While most rewarded outcomes resulted in a decrease in ACh, some trials showed characteristic biphasic patterns (pause-burst or burst-pause). Comparison with recordings of signals under the same behavioral conditions from ACh insensitive tdTomato, expressed in the same region, shows that this variability is not from measurement noise. **b**,**c**, Mean ACh (z-scored ΔF/F) signals aligned to reward (**b**) and no-reward (**c**) onset across all imaged mice for trials in early (black) and late (red) learning (reward trials: early = 206, late = 291; no-reward trials: early = 94, late = 134). Heatmaps of mean normalized responses to randomly selected trials (sorted by the time of minimum ACh dip) are shown at the bottom.

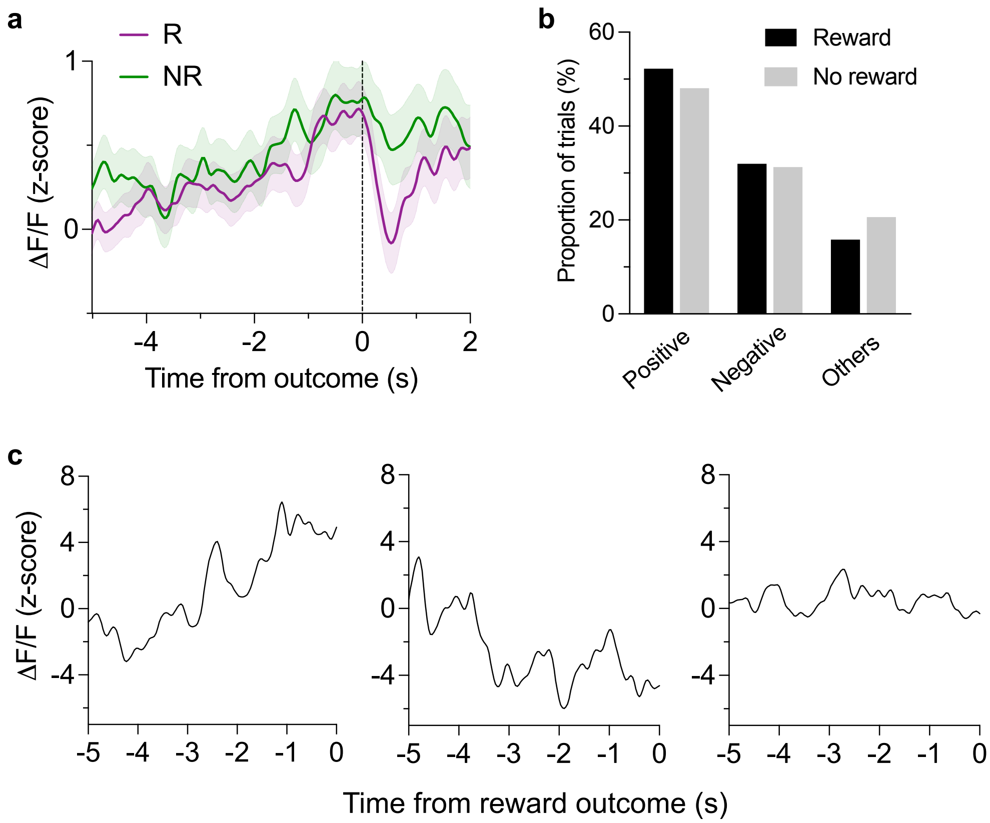

Supplementary Fig. 3: Dynamics of striatal acetylcholine signaling during goal approach. a, ACh fluorescence traces in the dorsal striatum aligned to goal outcome. Traces are grouped by trial outcome: rewarded (purple) and unrewarded (green) across n = 11 mice. **b**, Proportion of trials exhibiting distinct ramping profiles—positive, negative, or non-ramping ("other")—in the 5 seconds preceding trial outcome. Trial classification was based on linear regression analysis of ACh signals during this window; trials with a significant positive or negative slope ($P < 0.05$) were categorized accordingly, while non-significant traces ($P > 0.05$) were classified as "other." **c**, Example ACh signal profiles corresponding to each ramping category defined in **b**: positive ramp (left), negative ramp (middle), and non-ramping ("other") traces (right), aligned to trial outcome. Source data are provided as a Source Data file.

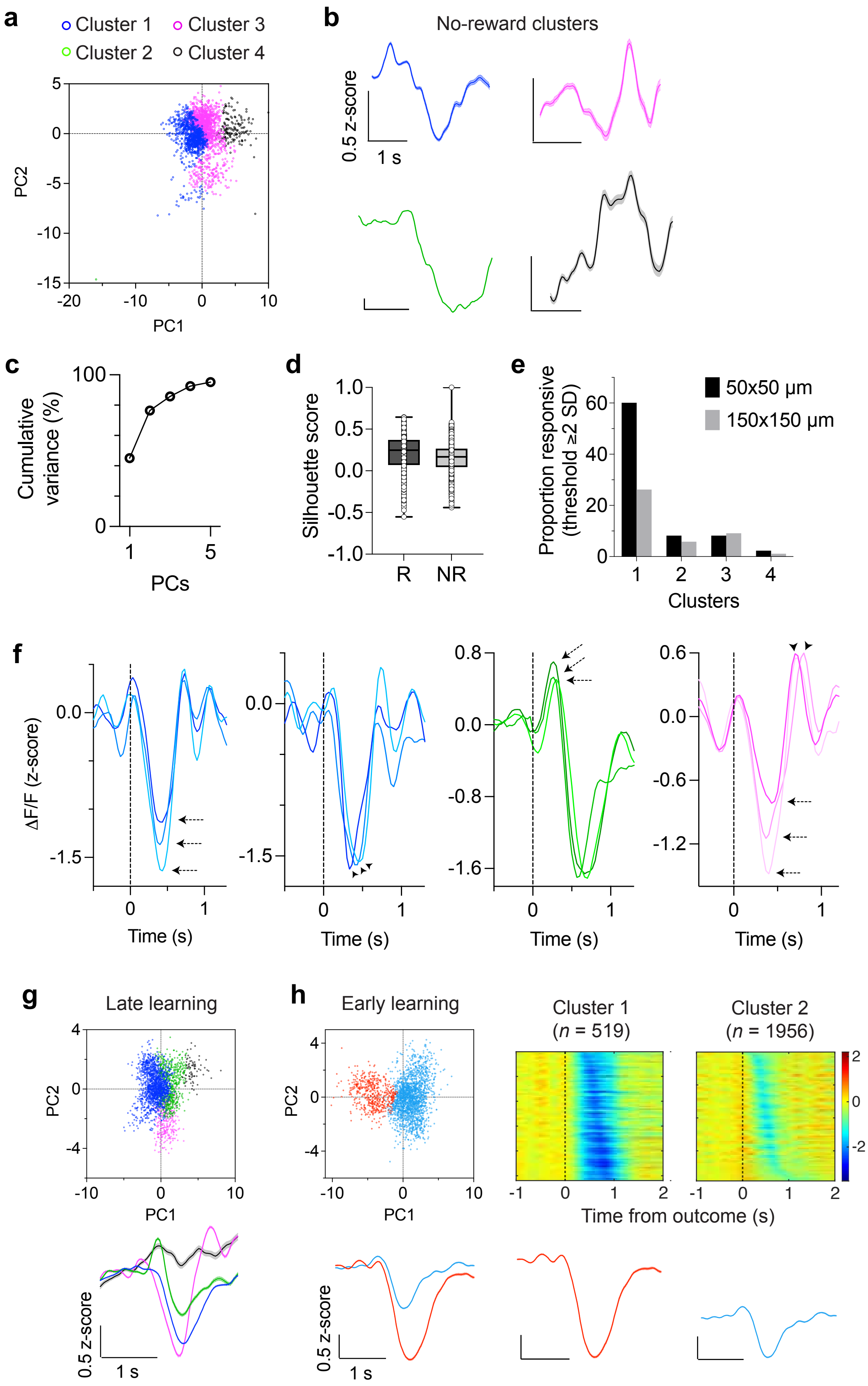

a
Cluster 1
Cluster 3
Cluster 2
Cluster 4
PC2
PC1
b
No-reward clusters
0.5 z-score
1 s
c
Cumulative variance (%)
PCs
d
Silhouette score
R
NR
e
Proportion responsive (threshold ≥2 SD)
50x50 µm
150x150 µm
Clusters
f
ΔF/F (z-score)
Time (s)
g
Late learning
h
Early learning
Cluster 1 (n = 519)
Cluster 2 (n = 1956)
Time from outcome (s)

Supplementary Fig. 4: Hierarchical clustering and temporal patterns of feedback responses. **a**, 2D score plot of the first two principal components (PC1 and PC2) of no-reward clusters. Colored circles indicate the four identified clusters defined based on hierarchical clustering. **b**, Mean iAChSnFR transients of all clusters with significant responses. **c**, Cumulative plots of the percent variance explained by the PCs shown against PC1 to PC5. **d**, Silhouette scores for hierarchical clustering of quadrat responses under reward and no-reward conditions. Silhouette analysis was used to evaluate the distinctness of clusters derived from hierarchical clustering of ACh spatiotemporal dynamics. The silhouette scores were 0.21 for reward and 0.22 for no reward, indicating that the identified clusters are not well-separated, but rather lie along a continuum of activity profiles. These scores suggest that quadrat responses exhibit gradual transitions rather than discrete response categories. **e**, Proportion of significant quadrats as a function of quadrat grid size (50×50 µm vs. 150×150 µm). This figure compares the percentage of quadrats with signal-to-noise ratios (SNRs) exceeding a 2SD threshold across two quadrat sampling resolutions. A greater proportion of significant quadrats was observed at the 50×50 µm grid size compared to the 150×150 µm grid size. This analysis informed our choice to use smaller quadrats, reflecting a practical balance between maximizing sensitivity (SNR) and maintaining spatial sampling resolution. **f**, Spatiotemporal patterns of example ACh quadrats in response to reward. Note the characteristic differences in response magnitude (dip amplitude, arrows) and temporal profiles (time to dip, or peak, arrow heads) of feedback responses. Some quadrats achieved reward-induced ACh dips (or peaks) at shorter latencies than others following reward delivery. Same color convention as Fig. 3b. **g,h,** Comparison of the 2D score plot of the first two principal components (PC1 and PC2) of identified clusters during the late (**g**) and early (**h**) phases of learning. Heatmaps depict the mean normalized responses of all clusters showing significant responses to reward in early learning. Individual quadrats are shown on y-axis and sorted by the time of their minimum ACh dip. Bottom traces represent population average within each cluster. Source data are provided as a Source Data file.

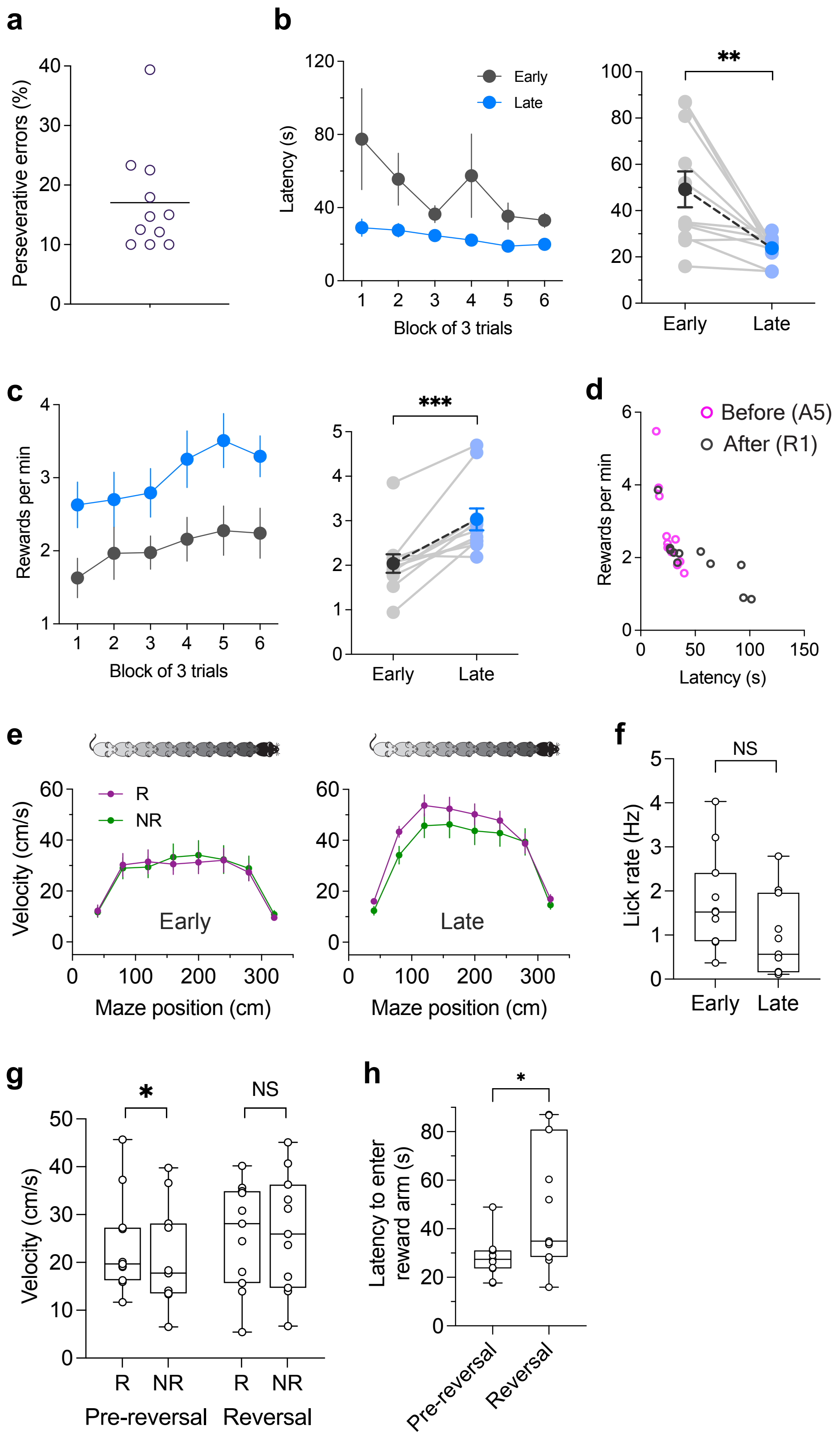
a
Perseverative errors (%)
b
Early
Late
Latency (s)
Block of 3 trials
**
c
Rewards per min
Block of 3 trials
***
d
Before (A5)
After (R1)
Rewards per min
Latency (s)
e
R
NR
Velocity (cm/s)
Early
Late
Maze position (cm)
f
NS
Lick rate (Hz)
g
*
NS
Velocity (cm/s)
R
NR
Pre-reversal
Reversal
h
*
Latency to enter reward arm (s)
Pre-reversal
Reversal

Supplementary Fig. 5: Mice behavioral performance during reversal learning. a, Percentage of perseverative errors (R1) across the population. Circles represent individual mice, line shows across-mouse mean (n = 11 mice). **b**, Left, Trial latency (time taken to complete a trial) broken down by trial block (first 6 block of trials, 3 trials/block). Right, Mean latency was significantly shorter in the late stage relative to the early stage (two-tailed, paired *t*-test, $P = 0.007$). **c**, Left, Reward rate broken down by trial block, as in **b**. Right, Mean reward rate was significantly higher in the late stage relative to the early stage (two-tailed, paired *t*-test, $P = 0.0005$). Transparent dots denote mean value from individual mice, and solid dots denote mean and S.E.M. across mice ($n = 11$). **d**, Mean latency vs reward rate. Reward rate (/min) became higher as mice completed trials at shorter latencies (s). **e**, Velocity (mean ± S.E.M) as a function of mice position in the maze (0-320 cm in 40-cm bins) during the early (left) and late phases (right) of reversal. **f**, There was a trend toward reduced anticipatory licking in the pre-outcome zone during approaches to the no-reward arm, from the early to late phases of reversal (two-tailed, paired *t*-test, $P = 0.15$). **g**, Approach velocity to the reward and no-reward arms differed significantly in the pre-reversal phase (two-tailed, paired *t*-test, $P = 0.02$) but not during the early phase of reversal (two-tailed, paired *t*-test, $P = 0.72$). **h**, Reward arm choices were associated with significantly longer trial durations post-reversal compared to pre-reversal trials (two-tailed, paired *t*-test, $P = 0.02$), possibly indicating increased deliberation or search-and-evaluate process in adaptive decision-making. Source data are provided as a Source Data file.

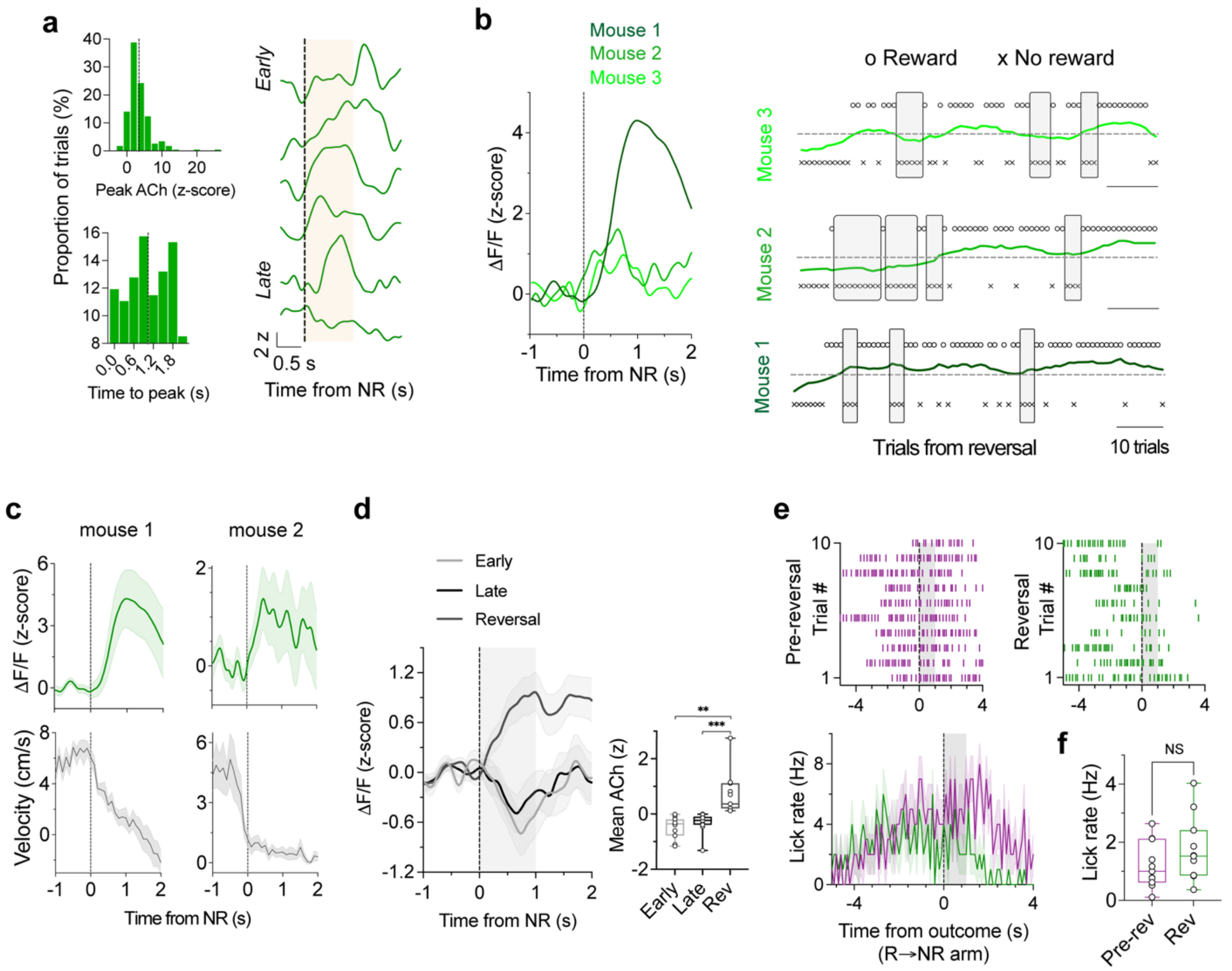

a
Proportion of trials (%)
Peak ACh (z-score)
Time to peak (s)
Early
Late
2 z
0.5 s
Time from NR (s)
b
Mouse 1
Mouse 2
Mouse 3
ΔF/F (z-score)
Time from NR (s)
o Reward
x No reward
Trials from reversal
10 trials
c
mouse 1
mouse 2
ΔF/F (z-score)
Velocity (cm/s)
Time from NR (s)
d
Early
Late
Reversal
ΔF/F (z-score)
Time from NR (s)
Mean ACh (z)
Rev
e
Pre-reversal
Trial #
Reversal
Lick rate (Hz)
Time from outcome (s)
(R→NR arm)
f
NS
Pre-rev

Supplementary Fig. 6: ACh responses to non-rewarded outcomes mediate reversal learning. **a** Left, distribution of the peak amplitude of ACh decreases (i.e., ACh peak, top) and mean latency of peak ACh activity (i.e., time-to-peak, bottom) following no-reward. Dashed black line represents the mean. Right, Example traces of single trial, spatially averaged (population) responses to no-reward outcomes. Dashed black line indicates no-reward event. **b**, Left, mean transient iAChSnFR signals aligned to NR following reversal in representative mice. Right, sample choice behavior around a reversal illustrating the trajectory of reversal learning for each mouse. Note a marked preference for the previously rewarded choice at the beginning of the reversal. Mice with stronger ACh responses to non-reward outcomes tended to exhibit frequent switching from unrewarded choices (gray shading). In contrast, mice with relatively weaker responses show sporadic (transient) shifts from unrewarded choices. "o" indicates rewarded and "x", unrewarded choices. Thick traces in the center indicate five-trial smoothing of choice behavior. **c**, Mean transient iAChSnFR signal (top) and velocity (bottom) aligned to time of NR following the reversal in two representative mice. Mice typically decelerated or stopped, with velocities approaching zero, during the same time window in which ACh levels increased. Dashed black line indicates no-reward event. **d**, Mean transient z-scored ΔF/F during outcome period of NR across learning (early, late, and reversal phases). Significant difference between conditions (early vs late, two-tailed, paired *t*-test, $P = 0.19$, early vs reversal, $P = 0.002$, late vs reversal, $P = 0.0007$, $n = 11$). **e**,**f**, Example anticipatory licking behavior from a representative mouse in randomly selected trials preceding (pre-reversal) and following contingency reversal (**e**). Anticipatory licking rate on approach to the previously rewarded arm was comparable to that for the now unrewarded arm following reversal (**f**) (two-tailed, paired *t*-test, $P = 0.31$, $n = 11$), suggesting a cognitive (internal) state of reward expectation post-reversal. Source data are provided as a Source Data file.

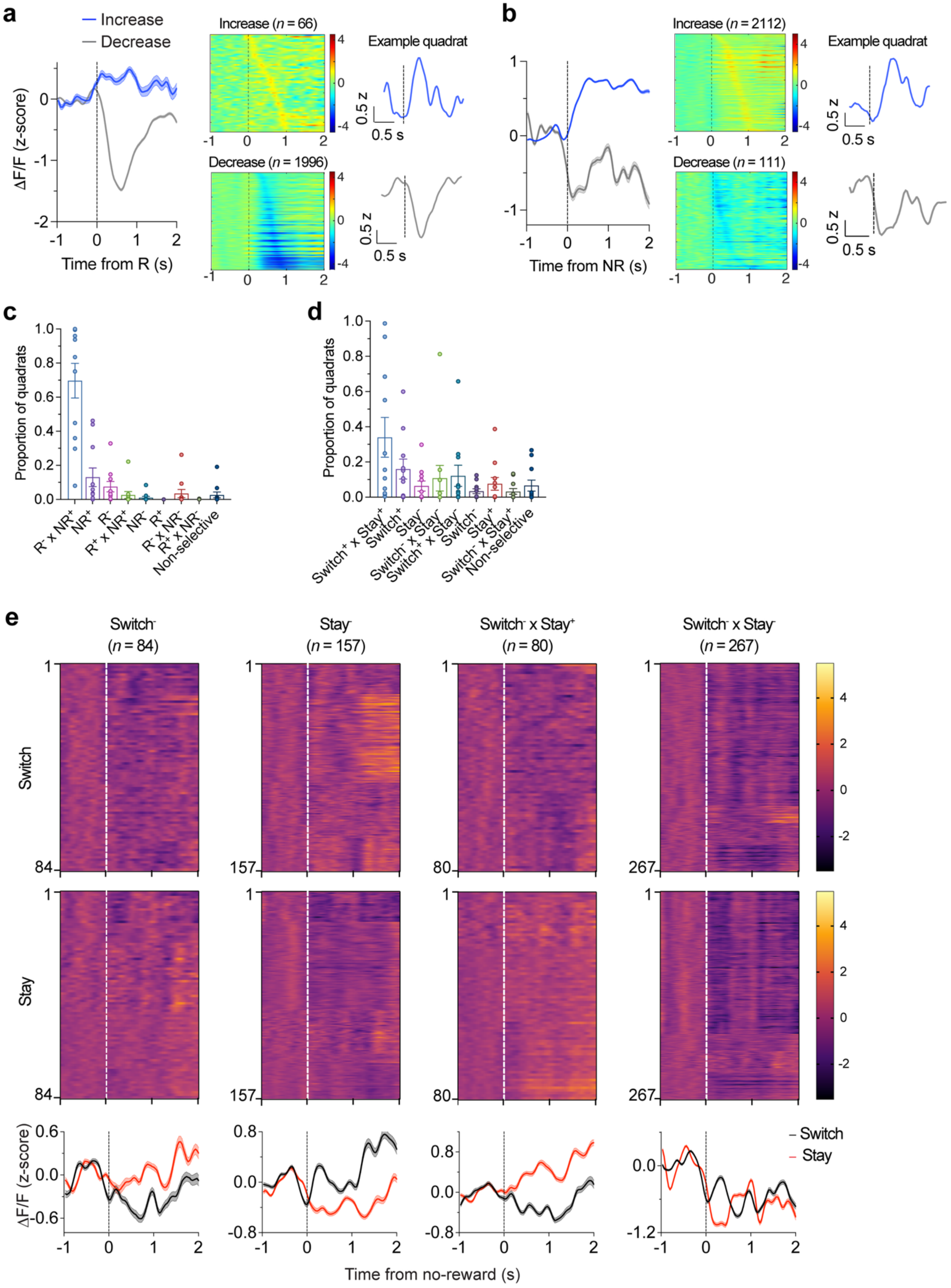

a
Increase
Decrease
ΔF/F (z-score)
Time from R (s)
Increase (n = 66)
Decrease (n = 1996)
Example quadrat
0.5 z
0.5 s
b
Time from NR (s)
Increase (n = 2112)
Decrease (n = 111)
Example quadrat
c
Proportion of quadrats
R⁻ x NR⁺
NR⁺
R⁻
R⁺ x NR⁺
NR⁻
R⁺
R⁻ x NR⁻
R⁺ x NR⁻
Non-selective
d
Proportion of quadrats
Switch⁺ x Stay⁺
Switch⁺
Stay⁻
Switch⁻ x Stay⁻
Switch⁺ x Stay⁻
Switch⁻
Stay⁺
Switch⁻ x Stay⁺
Non-selective
e
Switch⁻ (n = 84)
Stay⁻ (n = 157)
Switch⁻ x Stay⁺ (n = 80)
Switch⁻ x Stay⁻ (n = 267)
Switch
Stay
ΔF/F (z-score)
Switch
Stay
Time from no-reward (s)

Supplementary Fig. 7: ACh signaling of choice outcomes and future behavioral responses. a,b, Mean ACh signals of all responsive quadrats aligned to outcome onset following reversal. Mean across reward (a) and no-reward (b) trials. Quadrats were grouped on the basis of whether they showed significant increases or decreases to reward (increase, $n = 66$ quadrats, decrease, $n = 1996$), or no-reward (increase, $n = 2112$ quadrats, decrease, $n = 111$). Heatmaps depict mean normalized responses of all quadrats showing significant responses. Individual quadrats are shown on y-axis and sorted by the time of their maximum (top) or minimum (bottom) ACh activity. Example quadrat in each group is shown on the far right. Significance level was set to $P < 0.05$. **c**, Proportions of quadrats per mouse selectively responsive to various combinations of R and NR. Significance level was set to $P < 0.05$. **d**, Proportions of quadrats per mouse that encoded various combinations of future behavioral responses. **e**, Heatmaps of mean normalized responses of task-responding quadrats in **e**. Mean activity of individual quadrats are shown on the y-axis. Each row in the heatmaps represents responses from the same quadrat to future switch (top) or stay (bottom). Traces at the bottom represent population average within each cluster. Source data are provided as a Source Data file.

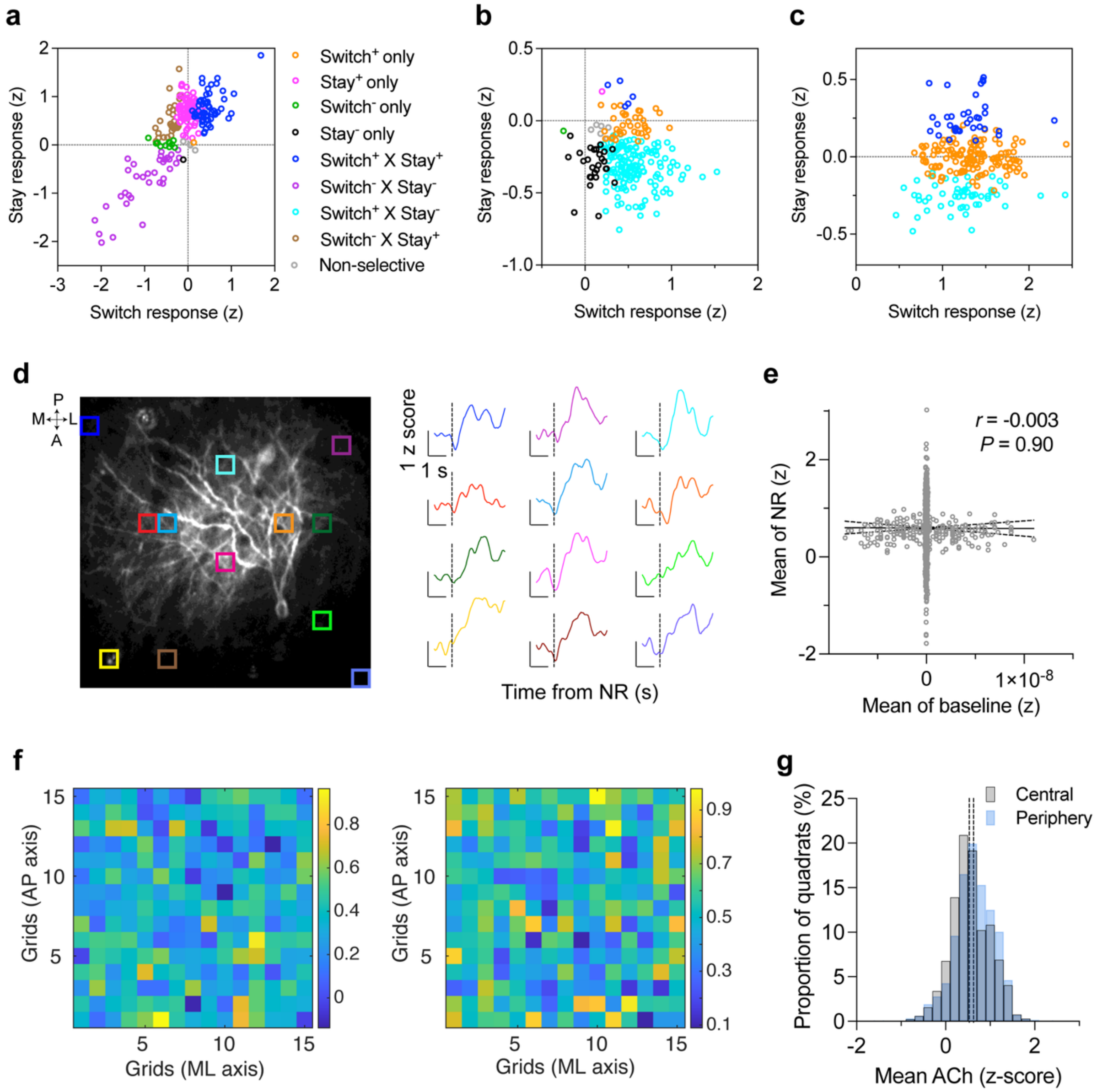

a
b
c
d
e
f
g
Stay response (z)
Switch response (z)
Switch⁺ only
Stay⁺ only
Switch⁻ only
Stay⁻ only
Switch⁺ X Stay⁺
Switch⁻ X Stay⁻
Switch⁺ X Stay⁻
Switch⁻ X Stay⁺
Non-selective
P
M
L
A
1 z score
1 s
Time from NR (s)
r = -0.003
P = 0.90
Mean of NR (z)
Mean of baseline (z)
1×10⁻⁸
Grids (AP axis)
Grids (ML axis)
Central
Periphery
Proportion of quadrats (%)
Mean ACh (z-score)

Supplementary Fig. 8: Spatiotemporal organization of ACh responses following reversal. **a-c**, Scatterplot comparing the distribution (clusters) of trial-averaged responses in the DS of three example mice. Each color represents a cluster, and each dot represents the normalized z-scored ΔF/F response of a quadrat preceding future behavior. Dotted lines represent the boundary between positive and negative responses on both axes. **d**,**e**, Mean fluorescence projection image for an example mouse during a representative behavior period post-reversal. Colored grids represent the spatial distribution of single quadrat responses shown on the right (**d**). The signal drop-off at the image periphery primarily reflects time-averaged baseline fluorescence and does not directly indicate a lack of behavior-linked activity. For analysis, quadrats were selected based on functional responsiveness, not by expression strength or spatial position alone, as the mean intensity projection may underrepresent transient or behavior-specific activity that occurred during particular epochs. Note the robust mean z-score activities even in regions with lower overall brightness. There was no significant correlation between the mean of the behavior-linked signals and baseline fluorescence across quadrats (**e**) (Pearson's r = -0.003, P = 0.90, simple linear regression, n = 2475 quadrats from 11 mice). **f**,**g**, Mean z-score heatmap of quadrats mapped onto a 15×15 grid across the imaging field in two representative mice post-reversal (**f**), with quadrats classified as peripheral (grids 1-2 and 14-15) and central areas (grids 3-13) based on radial distance from the imaging center. Note the overlap in distributions (**g**), suggesting that quadrats in peripheral and central areas show similar patterns of behavioral modulation across mice (n = 2475 quadrats across 11 mice). Dashed black lines denote the median. Source data are provided as a Source Data file.

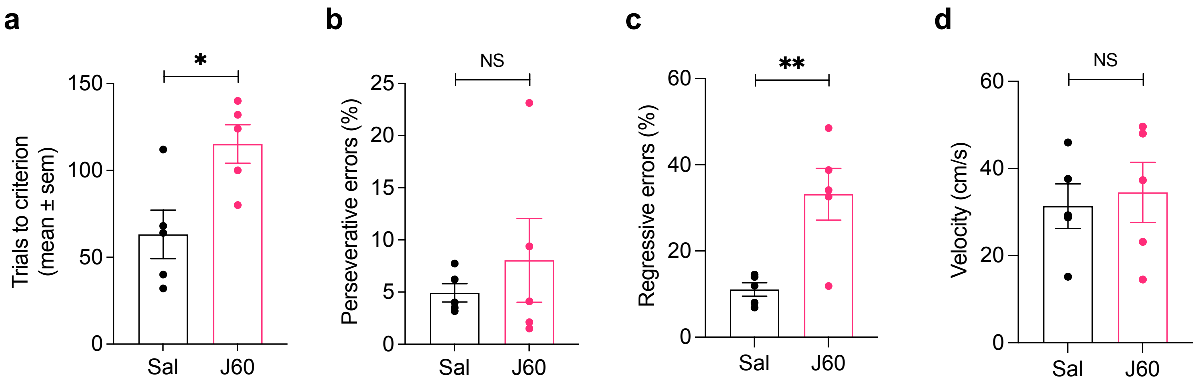

Supplementary Fig. 9: Causal role of CIN inhibition in reversal learning. a, Mean number of trials needed to reach criterion following reversal (two-tailed, unpaired *t*-test, $P = 0.019$, Cohen's $d = 1.67$, 95% CI [0.19, 3.15], $n = 5$ mice per group). **b**,**c**, Percentage of perseverative (b) and regressive (c) errors committed during reversal phase. CIN inhibition significantly increased regressive errors (two-tailed, unpaired *t*-test, $P = 0.007$, Cohen's $d = 2.04$, 95% CI [0.45, 3.62]) but not perseverative errors ($P = 0.47$, Cohen's $d = 0.43$, 95% CI [-0.82, 1.69]. **d**, There was no effect on mean run velocity (two-tailed, unpaired *t*-test, $P = 0.72$, Cohen's $d = 0.21$, 95% CI [-1.03, 1.45]). Data are mean ± S.E.M. Source data are provided as a Source Data file.

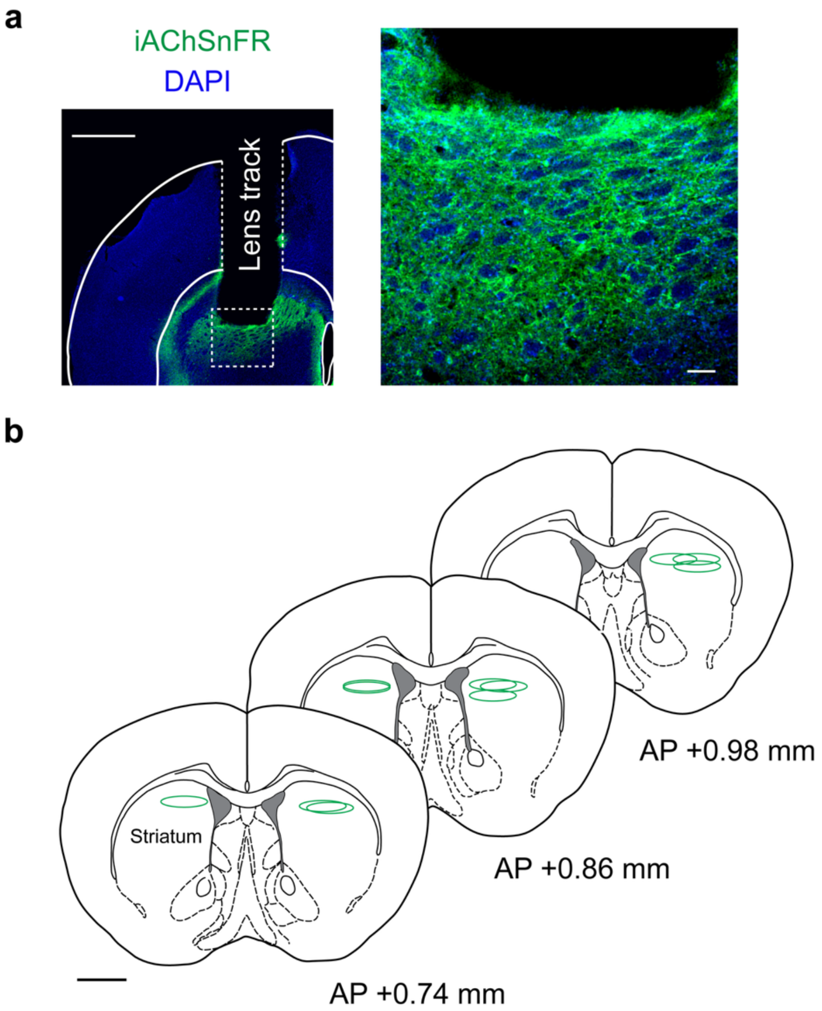

Supplementary Fig. 10: iAChSnFR expression and histological verification of GRIN lens placement. a, Example histology (left) showing immuno-enhanced iAChSnFR sensor expression and the GRIN lens track in a coronal section. Inset is shown on the right. Confocal image of immuno-enhanced iAChSnFR expression. Note the diffuse expression of the sensor on somatic membranes, dendrites, and neuropil. Scale bar, 1 mm (left) and 50 µm (right). Similar results were obtained in n = 10 other brains recovered for post-hoc validation of iAChSnFR sensor expression. **b**, Schematic showing the histologically verified locations of GRIN lens implants in the dorsal striatum of all animals used in the study. GRIN lens positions are plotted on the nearest reference slice at three different anterior-posterior levels relative to bregma of the atlas image[1]. Scale bar, 1 mm.

References

1. Franklin KDJ, Paxinos G. *The mouse brain in stereotaxic coordinates*, 3rd edn. Elsevier (2008).
